# Effect of the Filler Modification on the Thermal and Mechanical Properties of Composite Polypropylene/Wollastonite Drawn Fibers

**DOI:** 10.3390/polym15142986

**Published:** 2023-07-08

**Authors:** Konstantinos Leontiadis, Dimitris S. Achilias, Ioannis Tsivintzelis

**Affiliations:** 1 Department of Chemical Engineering, Aristotle University of Thessaloniki, 54124 Thessaloniki, Greece; 2 Department of Chemistry, Aristotle University of Thessaloniki, 54124 Thessaloniki, Greece

**Keywords:** polypropylene, wollastonite, modification, fiber, drawing

## Abstract

Polypropylene (PP) is one of the most commercially used thermoplastics, while a significant amount of PP is used in the form of fibers. In this study, the effects of modification of the filler on the thermal and mechanical properties of composite polypropylene/wollastonite drawn fibers were investigated. In this direction, the surface modification of wollastonite with various organic acids, such as myristic, maleic, malonic glutaric, pimelic, and suberic acid, and the use of two solvents were studied. The surface-modified wollastonite particles were used to produce composite polypropylene drawn fibers. The modification efficiency was found to be slightly better when a non-polar solvent (carbon tetrachloride) was used instead of a polar one (ethanol). FTIR experiments showed that myristic, maleic, malonic, and pimelic acid can strongly interact with wollastonite’s surface. However, the mechanical strength of the composite fibers was not increased compared to that of the neat PP fibers, suggesting inadequate interactions between PP and wollastonite particles. Furthermore, it was observed that the drawing process increased around 10% the crystallinity of all samples. Wollastonite modified with malonic acid acted as a nucleating agent for *β*-crystals. The onset decomposition temperature increased by 5–10 °C for all samples containing 2% wollastonite, either modified or not. The suggested modifications of wollastonite might be more suitable for less hydrophobic polymers.

## 1. Introduction

Polyolefins consist of the most economically important family of polymers. Globally, the most produced polyolefins are polyethylene (PE) and polypropylene (PP) [1]. Isotactic PP presents higher melting temperatures compared to PE, low density, and chemical inertia and is a versatile material that allows processing in various forms, i.e., dense solid parts, films, and fibers. However, about one-third of PP’s annual production is related to polymer fiber applications [2]. PP fibers are used as raw materials for fabric production (woven or non-woven) and also for the packaging, agriculture, and construction sector [3].

Isotactic polypropylene (iPP) is the most common commercial form of PP. It is a semicrystalline material presenting 40 to 70% crystallinity [4]. The most common crystals that are found in the iPP matrix are *α*-crystals. *β*-crystals are more rarely found and are produced under shear stress [5,6], using *β*-nucleating agents, or under specific crystallization conditions (e.g., by crystallizing iPP in the temperature range of 115–135 °C) [7]. *β*-crystals melt at lower temperatures and present lower density compared to *α*-crystals, while they may exhibit higher crystallization rates, depending on the crystallization temperature [8,9]. Upon heating, the conversion of *β*-crystals to *α*-crystals is observed. This effect is known as *βα*-recrystallization, which may also occur under tensile stress, e.g., during the process of drawing [7,10]. The existence of *β*-crystals in non-drawn samples facilitates the drawing process [10] and results in enhanced mechanical properties after drawing [11].

Polymer chain orientation obtained via the biaxial drawing of polymer films or the uniaxial drawing of polymer fibers has a considerable impact on various properties, such as crystallinity, tensile strength, and fragility at low temperatures [4]. Under appropriate process conditions, drawing can result in a tremendous increase in tensile strength. For example, a uniaxial drawing process with a drawing ratio equal to 7 may increase more than ten times the tensile strength of PP, from around 30–40 MPa to around 400–500 MPa [12,13].

Besides drawing, inorganic fillers are typically used to enhance the mechanical and thermal properties of PP. The drawing of polymer composite fibers containing needle-shaped additives (such as wollastonite or carbon nanotubes) tends to align the filler particles to the drawing axis parallel with the chain orientation, resulting in a further increase in mechanical properties [14,15]. In this direction, PP–single wall carbon nanotube composite drawn fibers, produced using a drawing ratio equal to 21, exhibited tensile strength up to 802 MPa [16]. Also, PP–wollastonite composite drawn fibers produced, using a drawing ratio of 7–9, exhibited a tensile strength of up to 527 MPa [13].

PP, as a polyolefin, is characterized by a non-polar and hydrophobic nature, contrary to the polar nature of common additives such as montmorillonite, talc, wollastonite, etc. Thus, intermolecular interactions of PP macromolecules with most inorganic additives are rather poor. In order to increase the favorable intermolecular interactions, the surface modification of the additive particles, the modification of the polymer matrix, or both of them are required [12]. Although one of these procedures may be enough for polar polymers, for PP, both of them are usually utilized [17,18,19]. The grafting of PP chains with polar groups, such as maleic anhydride, results in more thermodynamically favored interactions with hydrophilic fillers, which, in turn, may result in better dispersion of the filler particles inside the polymer matrix, causing a significant improvement of composite’s properties [12,19,20,21,22,23,24,25]. In this direction, using PP-g-MA as a compatibilizer in PP–wollastonite composites increased the yield strength of PP-silane modified wollastonite from 25.3 to 31.8 MPa [19].

Wollastonite is an inosilicate mineral that belongs to pyroxenoids. It is commonly found in the form of micro-sized needle-shaped particles. However, besides its natural occurrence, nano-sized wollastonite particles can be synthesized [26]. Wollastonite has been utilized as an additive for PP in order to enhance the mechanical properties [19,26,27,28,29,30,31,32,33], increase the crystallinity [28,29,30], and improve the thermal stability [26,34] of the polymer matrix. However, most of such literature studies do not refer to drawn or fibrous PP composites. Since drawing has a severe effect on mechanical properties, further increase of tensile strength by the addition of inorganic particles in composite drawn fibers is a difficult task and less studied compared to PP non-drawn composites. Consequently, despite the fact that around 30–35% percent of the produced wollastonite is used in polymer applications [35], there are only a few studies on PP–wollastonite composite drawn fibers [12,13,36], rendering the effect of the needle-shaped fillers on the alignment of polymer chains as an issue that requires further investigation.

As mentioned above, the surface modification of hydrophilic inorganic fillers is needed to improve their interactions with the PP matrix. Wollastonite modification is typically achieved via a reaction with carboxylic acids and, more specifically, via the interaction between the calcium ions present on the surface of wollastonite and the carboxyl group of organic acids [29,31,32,37,38]. In this direction, it has been observed that the dispersion of wollastonite in the PP matrix was improved by modifying the surface of wollastonite with malonic, pimelic, and stearic acid [29,32,37]. Besides the improvement of particle dispersion, such a surface modification of wollastonite alters the crystallization behavior of PP since wollastonite modified with malonic acid [32] or pimelic acid [29,31,38] has been reported to induce *β*-crystals formation. The same nucleating effect has been reported for calcium carbonate salts modified with malonic, glutaric, pimelic, and suberic acid, which showed a strong *β*-crystal nucleating effect for PP upon isothermal crystallization at 130 °C [39].

A factor that has not been thoroughly studied in the literature is the effect of organic solvents used for the surface modification of the filler. In literature studies, the solvents used for the modification of wollastonite with carboxylic acids are usually ethanol [29,32,40] and acetone [31,38,41]. Rao et al. used different types of solvents to modify wollastonite with stearic acid. In more detail, acidic, basic, and non-polar solvents were tested, and the authors concluded that non-polar solvents resulted in higher acid absorption on the surface of the particles, while the absorption decreased with increasing acidity or basicity of the solvents [40]. In some cases, hybrid processes to modify wollastonite have been investigated, such as grinding in the presence of organic acids [31,38,42,43].

Regarding the mechanical properties of composite PP–wollastonite materials (non-drawn materials), it has been reported that for wollastonite content up to 2.5 wt%, tensile strength was increased for composites containing modified wollastonite with pimelic acid, while filler contents higher than 2.5 wt% were found to have a negative effect on such property [29]. Also, for the same wollastonite content (2.5 wt%), the impact strength was considerably improved for the composites containing modified wollastonite with malonic acid, but no significant improvement was observed regarding the tensile strength [32].

Nevertheless, the findings mentioned above are related to non-fibrous and non-drawn materials. Thus, the aim of this work is to study the effect of the modification pathway (type of acid and solvent used for the modification) of wollastonite on the mechanical and thermal properties of PP/modified wollastonite composite drawn fibers.

## 2. Materials and Methods

### 2.1. Materials

Isotactic PP (Ecolen HZ42Q) with melt flow index equal to 18 g/10 min, tensile strength equal to 33 MPa, and melting point of 168–171 °C was obtained from Hellenic Petroleum S.A., Thessaloniki, Greece. A masterbatch (Bondyram^®^ 1001) with PP grafted with maleic anhydride (PP-g-MA), with MA content of 1%, melt flow index equal to 100 g/10 min, and melting point of 160 °C, obtained from Polyram Plastic Industries Ltd., Gilboa, Israel, was used as a compatibilizer. Wollastonite in the form of needle-like particles (NYAD M9000) with aspect ratio equal to 3 (9 μm length to 3 μm diameter) was kindly supplied by Imerys Minerals Ltd., Paris, France.

Two different organic solvents were used, namely, ethanol and carbon tetrachloride, for the modification procedure of wollastonite. Ethanol was purchased from Honeywell, Charlotte, NC, USA (purity ≥ 99.8%), while carbon tetrachloride was purchased from Fluka Chemie AG, Buchs, Switzerland (purity > 99.5%).

Myristic, malonic, glutaric, pimelic, and suberic acid were purchased from Sigma-Aldrich, St. Louis, MO, USA, while maleic acid was purchased from Alfa Aesar, Haverhill, MA, USA. Potassium bromide (KBr, >99.5%) was purchased from Chem-Lab, Zedelgem, Belgium and was dried at 170 °C for 3 h prior to use. All other chemicals were used as received (unless otherwise noted).

### 2.2. Wollastonite Modification

**Figure 1:**
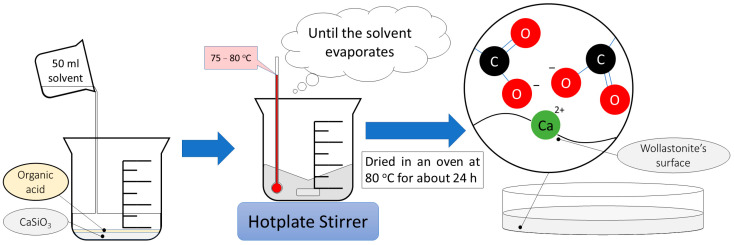
Modification of wollastonite with organic acids.

Prior to the modification process, pristine wollastonite was dried at 110 °C for 6–8 h in order to remove excess moisture. The dried wollastonite–organic acid mixture (10 g) and 50 mL of the solvent (ethanol or carbon tetrachloride) were added to a beaker, which was placed on a hotplate stirrer inside a fume hood. The modification took place at 80 °C until all the solvent was evaporated. Organic acids and wollastonite weighing were carried out using a Sartorius B 120 S scale (accuracy ± 0.1 mg). After the modification process, the obtained modified wollastonite was once more dried in an oven at 80 °C for about 24 h. Finally, the dried modified wollastonite was ground in powder form using a mortar. The modification process is shown in Figure 1.

### 2.3. Fiber Production and Drawing

Weightings of the polymer pellets and the wollastonite powder were carried out with a KERN PLS 1200—3A scale (±0.003 g). In all cases, pellets of isotactic polypropylene were mechanically pre-mixed with pellets of the compatibilizer’s masterbatch (PP-g-MA) and wollastonite in powder form, either modified or not. Initially, the mixture of PP and additives (compatibilizer and wollastonite) was inserted in a twin screw (screw rotating speed = 25 rpm) extruder (HAAKE Rheodrive 5001) with four heating zones (190, 210, 215, and 220 °C from feed to nozzle). The produced filament was cut into pellets and fed into a single-screw (screw rotating speed = 15 rpm) extruder (Noztec Xcalibur) with three heating zones (215, 225, and 210 °C from feed to nozzle). A winding machine (Noztek Filament Winder 2.0) was used to collect the produced fiber into a drum. Finally, solid state drawing was performed at 140 °C, keeping the drawing ratio constant and equal to 7. More details can be found in previous studies [12,13].

### 2.4. Characterization

Thermal properties of the samples were studied via differential scanning calorimetry (DSC) and thermogravimetric analysis (TGA) using Shimadzu DSC-50 and Shimadzu TGA-5 instruments, respectively. DSC measurements were performed with a heating rate of 10 °C/min up to 210 °C under nitrogen atmosphere, while TGA measurements were performed with a heating rate of 20 °C/min up to 550 °C under nitrogen atmosphere. Heat of fusion was calculated using the standard procedure. The degree of crystallinity was estimated by dividing the measured heat of fusion by the heat of fusion of 100% crystalline PP, which was considered equal to 209 J/g [44,45] and 185 J/g [10] for *α* and *β* form, respectively. The onset decomposition temperature was considered as the temperature at which the remaining mass is equal to 97% of the initial mass.

Mechanical properties were studied via tensile tests using Hans Schmidt & Co. GmbH (Waldkraiburg, Germany) Universal Testing Machine ZPM equipped with a Pacific PA6110 loadcell (headspeed 100 mm/min). For each sample, at least 15 tensile tests were performed, and the values presented in the next sections are the average values of all measurements. It should be noted that in our previous studies [12,13,16], the strain (reduced elongation) was expressed as the length of the specimen divided by the initial length (l/l_o_), while in this study, the strain is expressed as the length of the specimen minus the initial length divided by the initial length ((l−l_o_)/l_o_).

Lastly, Fourier Transform Infrared (FTIR) spectroscopic measurements were performed in KBr pellets using a Biorad FTS 175 spectrometer. For each sample, 64 scans were collected in the range of 400–4000 cm^−1^ with a resolution of 2 cm^−1^. KBr pellets were prepared by mixing the sample and KBr in 1:200 mass proportion and grinding in a mortar in order to obtain a fine powder. The powder was processed into pellets in a hydraulic press (applying pressure equal to 100 bar).

## 3. Results and Discussion

In the next subsections, firstly, the effect of the compatibilizer on the final properties of the produced fibers is studied. Then, the effect of the solvent used for the modification of wollastonite is explored. Finally, the effect of different organic acids on the modification of wollastonite and the final thermal and mechanical properties of the composite PP drawn fibers are presented.

### 3.1. Effect of the Compatibilizer

In this subsection, the effect of compatibilizer in the composite drawn fibers is discussed. Eight samples were prepared, as shown in Table 1. For their preparation, wollastonite was modified using myristic acid, and ethanol was used as a solvent. Their names, shown in Table 1, indicate their composition as the first number is related to the percentage of myristic acid used for the wollastonite modification, the second number indicates the percentage of wollastonite content in the composite material, and, lastly, NoBR is added to the end of the sample name, if compatibilizer was not used in this sample. The characterization results are shown in Table 2. Typical stress–strain, DSC (before drawing and after drawing), and TGA curves are shown in Figures S1–S4, respectively, of the Supplementary Information file.

As mentioned in Section 2.4, for each sample, at least 15 tensile tests were performed. The standard deviation of such measurements is indicated by the ±values in Table 2. Since the accuracy of the method is at least one order of magnitude lower than the reported deviations, those deviations are mainly attributed to the heterogeneity of the polymer matrix due to non-uniform drawing and/or particle dispersion. However, in both cases (with and without the use of compatibilizer), the lower deviations in Young’s modulus and tensile strength, thus the better uniformity of the polymer matrix, are observed for the highest modification ratio (2 g of myristic acid per 1 g of wollastonite). Furthermore, samples containing compatibilizer seem to exhibit higher Young’s modulus and slightly higher tensile strength compared to the relevant samples prepared without its addition, although in both cases, the differences are smaller than the standard deviation of such measurements. It is apparent that by increasing the modification ratio, the tensile strength is decreasing.

All samples presented only *α*-crystals, as the only peak that appeared in the DSC thermogram was observed around 160 °C. Furthermore, from the results of Table 2, it is concluded that the drawing process significantly increases the crystallinity of the fibers. Similar results have been reported for pure PP fibers obtained using lower drawing ratios than the ones used in this study [46]. Moreover, before drawing, the wollastonite-containing samples (1_2, 2_2, 1_2_NoBR, and 2_2_NoBR) present higher crystallinity than the samples without wollastonite (0_0 and 0_0_NoBR). This effect can be attributed to the nucleating effect of wollastonite [12]. Lastly, there is no clear conclusion regarding the effect of the compatibilizer on the crystallinity of the samples.

The onset decomposition temperature shown in Table 2, considering the temperature of 97% remaining mass, increases with the addition of wollastonite (either modified or not). Similar results have been reported in the literature. In more detail, composite PP fibers with 4 wt% wollastonite, 5.1 wt% compatibilizer, and prepared using a drawing ratio equal to 9 exhibited onset decomposition temperatures around 300 °C [13], while samples without additives showed lower values (around 270 °C) [12]. Furthermore, the onset decomposition temperature seems to increase by increasing the wollastonite’s modification percentage, at least for the samples that contain compatibilizer. This should be attributed to the above-mentioned better dispersion of the filler.

Based on such results, compatibilizer was added in all samples that are studied in the next sections.

### 3.2. Effect of the Solvent

In this section, the effect of the solvent on the modification of wollastonite is presented. Two solvents were studied: ethanol, which is a polar solvent used in most similar literature studies [29,32,40], and carbon tetrachloride, which was found to enhance the adsorption of steric acid in wollastonite, compared to acidic or basic solvents [40]. In all cases, myristic acid was used as a modifier. Details about the prepared wollastonite samples, prepared according to the procedure described in Section 2.4, are presented in Table 3.

**Figure 2:**
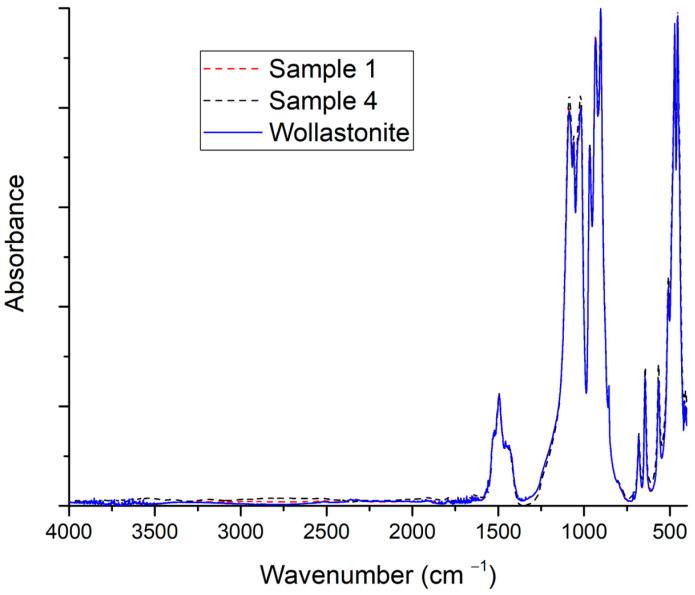
FTIR scans of samples 1 and 4 (see Table 3) and comparison with the scan of pristine wollastonite. No organic acid was used for the modification of samples 1 and 4.

In Figure 2, the FTIR analysis results of samples 1 and 4 (see Table 3) are shown. Such wollastonite samples were treated (with the solvents) in a similar manner to the modification procedure but without the addition of acid. They were prepared to reveal any potential permanent effect of the solvent on the wollastonite surface. From Figure 2, it is apparent that both solvents, ethanol and carbon tetrachloride, do not alter the surface of wollastonite since both FTIR scans are practically identical to the scan of the pristine wollastonite sample.

**Figure 3:**
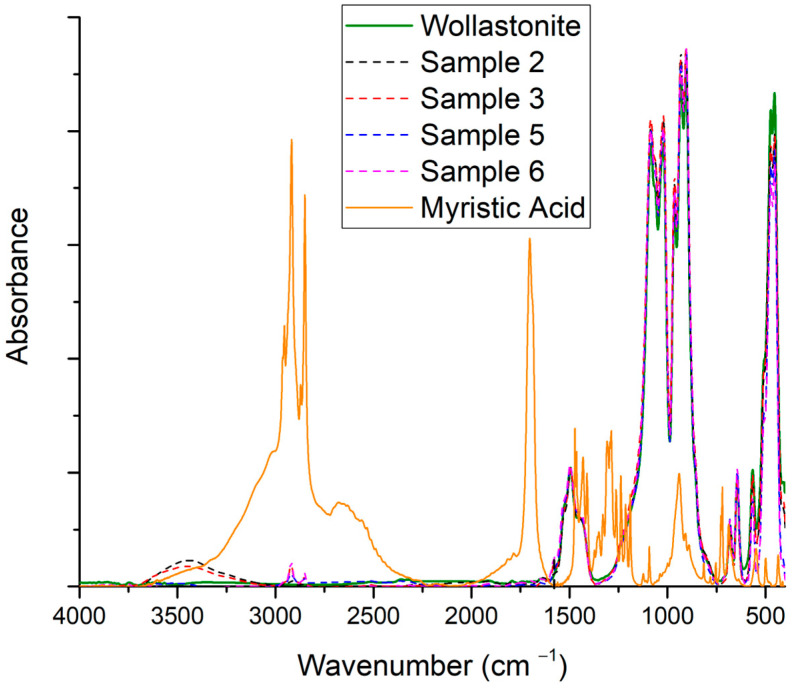
FTIR scans of samples 2, 3, 5, and 6 (see Table 3), as well as scans of myristic acid and pristine wollastonite.

**Figure 4:**
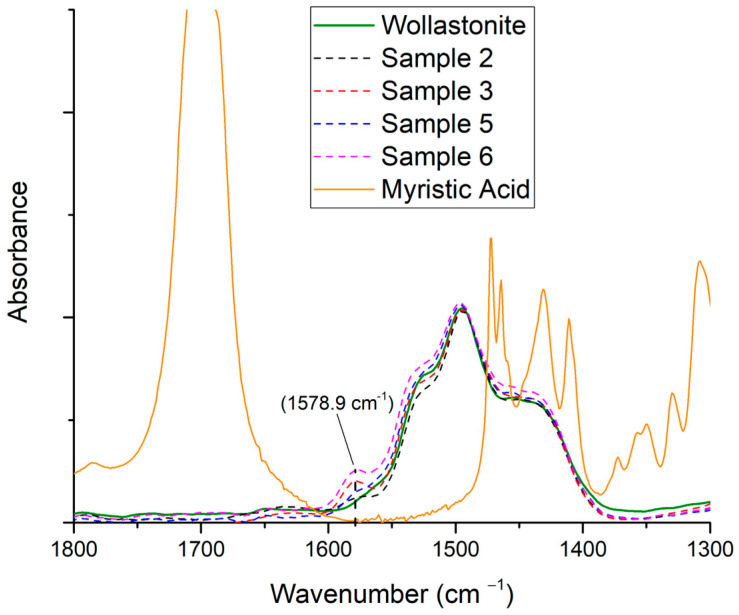
FTIR scans of samples 2, 3, 5, and 6 (see Table 3), as well as scans for myristic acid and pristine wollastonite (detail of Figure 3).

In Figure 3, the FTIR scans of samples 2, 3, 5, and 6 are presented, along with the scans of myristic acid and pure wollastonite. Samples 2 and 3 exhibit a broad peak around 3500 cm^−1^, which could be attributed to moisture and/or traces of ethanol. Three significant changes in modified samples can be observed in Figure 3. Firstly, the peak at 1700 cm^−1^ (stretch of C=O of the carboxylic group), which is apparent in myristic acid, is not visible in the modified samples. Also, new peaks around 1580 cm^−1^ are observed in the modified samples (the peaks are more easily realizable in Figure 4). This absorption band is related to carboxylate anion (COO^−^) stretching [2,32]. According to Meng and Dou, the appearance of such a peak (at 1580 cm^−1^) indicates that the –COOH of the organic acid reacted with the Ca^2+^ on the surface of wollastonite [29], suggesting that the organic acid was chemically adsorbed and formed a non-extractable layer on the wollastonite’s surface [29,32].

In order to quantitatively compare the effect of the two solvents on the modification of the wollastonite surface, the area of the new peak (at 1580 cm^−1^) was divided by the area of three characteristic wollastonite peaks, and the results are shown in Table 4 (calculation of relative absorbance). It should be noted that such relative absorbance has been divided by the content of myristic acid of each sample (1.974 g of myristic acid per 1 g of wollastonite and 2.028 g of myristic acid per 1 g of wollastonite for samples 3 and 6, respectively). The calculations were performed for samples 3 (for ethanol) and 6 (for carbon tetrachloride) since, in these samples, the new peak was more intense.

For all three peak ratios in Table 4, the relative absorbance is higher for the sample modified in carbon tetrachloride compared to the sample modified in ethanol. Though the difference is rather low, it is a reasonable result. More precisely, ethanol can react with organic acids (via the esterification reaction), decreasing the available organic acid molecules that could interact with wollastonite’s surface. On the other hand, carbon tetrachloride is a non-polar solvent that does not react with organic acids. Similar results have been reported by Rao et al. [40].

Each wollastonite sample (see Table 3) was used for the preparation of composite drawn fibers, and their mechanical and thermal properties were investigated. The composition of such fibers is shown in Table 5. All samples contained 4% compatibilizer and 2% wollastonite. The name of each sample is an indicator of its composition. The number indicates the wollastonite’s modification percentage, while “Eth” or “CCl_4_” indicate the solvent used for the modification. The prepared PP–wollastonite composite drawn fibers were characterized using tensile tests, DSC, and TGA analysis, and the results are presented in Table 6. Samples 0_Eth, 1_Eth, and 2_Eth are the same as 0_2, 1_2, and 2_2 from the previous section. The names of the samples were changed for easier comparison among the solvents. Typical stress–strain, DSC before drawing and after drawing, and TGA curves are shown in Figures S5–S8, respectively, of the Supplementary Information file.

Similarly to the results presented in the previous section (Section 3.1, Effect of the compatibilizer), the standard deviation of the experimental tensile strength values, indicated by the ±values of Table 6, is the lowest for the highest investigated myristic acid to wollastonite ratio (see samples 2_Eth and 2_CCl_4_). Using ethanol as a solvent, a significant decrease in tensile strength is observed with increasing the used myristic acid, while in the case of CCl_4_, the highest tensile strength is observed for the 1 g/1 g acid to clay ratio. However, no clear conclusion can be made since the differences in the mean values are within the standard deviation of such measurements. Also, it is confirmed that the drawing process induces the formation of crystals and increases crystallinity. Moreover, the solvent type does not seem to significantly affect the crystallinity of the composite fibers, either drawn or not. Lastly, the TGA results are similar to the results discussed in the previous section, i.e., the onset decomposition temperature increases with increasing the modification ratio (myristic acid to wollastonite), a result that should be attributed to the better dispersion of filler.

From the results presented in this section, it seems that there are no significant differences in the properties of the composite fibers, which were prepared using ethanol or carbon tetrachloride for the modification of wollastonite. However, since the use of carbon tetrachloride results in more pronounced acid adsorption, as shown by the results of Table 4, all subsequent samples, studied in the next section, were prepared with wollastonite that was modified using carbon tetrachloride as a solvent.

### 3.3. Effect of Organic Acid Used for Wollastonite’s Surface Modification

In this section, the effect of various organic acids used for the modification of wollastonite’s surface is investigated, and the properties PP–modified wollastonite composite drawn fibers are presented and discussed. Six different organic acids were used to modify wollastonite’s surface at three different ratios, as presented in Table 7. One aliphatic monocarboxylic acid (myristic acid), four aliphatic dicarboxylic acids (malonic acid, glutaric acid, pimelic acid, and suberic acid), and one aliphatic unsaturated dicarboxylic acid (maleic acid) were used. Myristic acid was already used in the study of the compatibilizer and solvent effect (see previous sections).

Three ratios of organic acid moles per wollastonite grams were used. Moles of organic acid per gram of wollastonite were used instead of grams of organic acid per gram of wollastonite in order to assure that a comparable number of carboxylic groups were used in all cases.

#### 3.3.1. Myristic Acid

**Figure 5:**
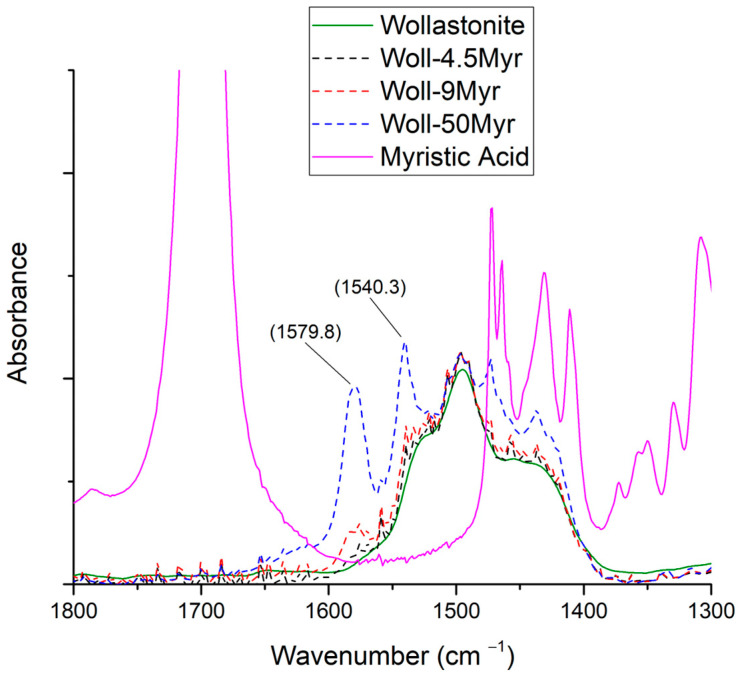
FTIR spectra of myristic acid, wollastonite, and modified wollastonite with myristic acid in three ratios.

In Figure 5, the FTIR spectra of myristic acid, wollastonite, and wollastonite modified with myristic acid at three different ratios are shown in the 1800–1300 cm^−1^ range. The full spectra, at the 400–4000 cm^−1^ range, are shown in Figure S9 of the Supplementary Information file. In the spectrum of the modified wollastonite, a new peak has appeared around 1580 cm^−1^. Moreover, a new peak at 1540 cm^−1^ is observed for the sample with the highest acid-to-wollastonite ratio. Both new peaks are connected to the stretching of the COO^−^ group [47]. In addition, the characteristic peak of acids around 1700 cm^−1^ is not visible in the modified wollastonite samples. As mentioned in Section 3.2, the appearance of such peaks at 1580 cm^−1^ and 1540 cm^−1^ indicates that the –COOH of the organic acid interacted with the Ca^2+^ on the surface of wollastonite [29]. Consequently, the above observations indicate a successful modification of wollastonite’s surface.

#### 3.3.2. Maleic Acid

**Figure 6:**
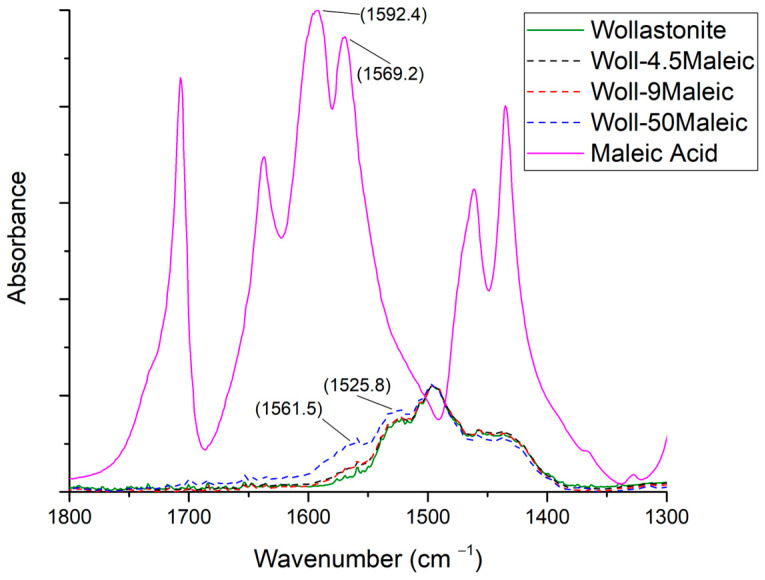
FTIR spectra of maleic acid, wollastonite, and modified wollastonite with maleic acid in three ratios.

In Figure 6, the spectra of maleic acid, wollastonite, and wollastonite modified with maleic acid at three ratios are shown in the 1800–1300 cm^−1^ range. The full spectra, at the 400–4000 cm^−1^ range, are shown in Figure S10 of the Supplementary Information file. The only undoubted difference is observed for the sample with the highest acid-to-wollastonite ratio. More specifically, in Figure 6, two new peaks are observed around 1525 and 1560 cm^−1^. These peaks can be interpreted as shifted peaks already existing in pure maleic acid at 1570 and 1590 cm^−1^. These peaks in pure maleic acid are attributed to the stretching of COO^−^, as maleic acid creates intramolecular hydrogen bonds [48]. Such shifted peaks in the modified wollastonite surface suggest the existence of some interaction between acid and wollastonite.

#### 3.3.3. Malonic Acid

**Figure 7:**
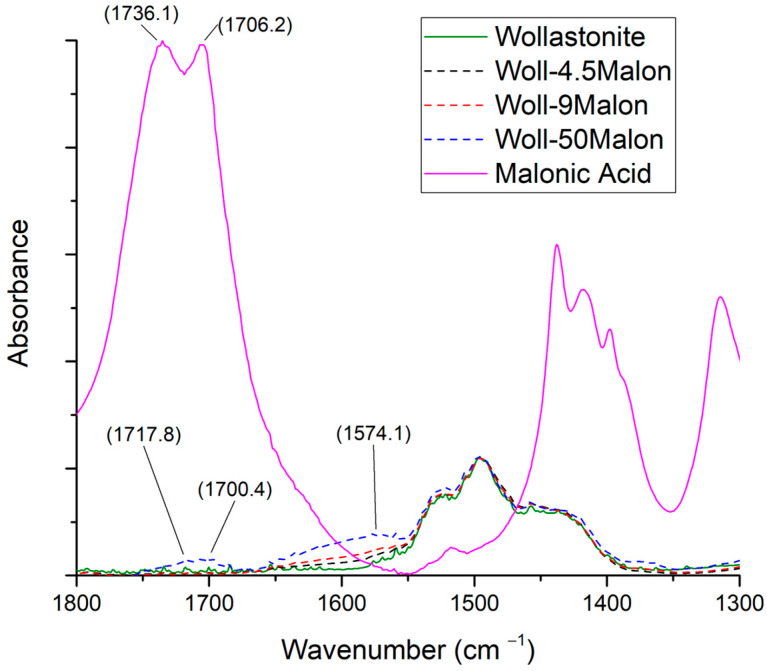
FTIR spectra of malonic acid, wollastonite, and modified wollastonite with malonic acid in three ratios.

In Figure 7, the spectra of malonic acid, wollastonite, and wollastonite modified with malonic acid at three ratios are shown in the 1800–1300 cm^−1^ range. The full spectra, at the 400–4000 cm^−1^ range, are shown in Figure S11 of the Supplementary Information file. In the case of malonic acid, the peak around 1580 cm^−1^, attributed to the stretching of COO^−^, becomes stronger as the malonic acid to wollastonite ratio increases.

#### 3.3.4. Glutaric Acid

**Figure 8:**
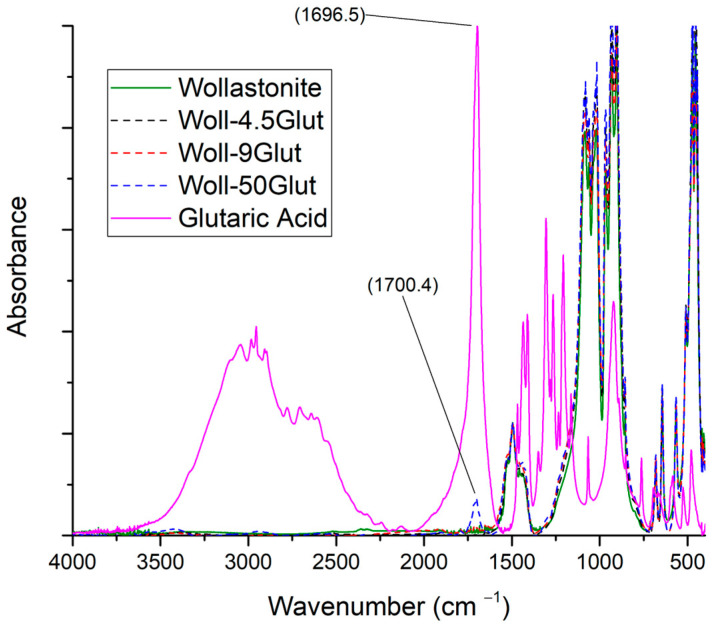
FTIR spectra of glutaric acid, wollastonite, and modified wollastonite with glutaric acid in three ratios.

In Figure 8, the spectra of glutaric acid, wollastonite, and wollastonite modified with glutaric acid at three ratios are shown. There is no visible difference among the spectra of wollastonite and modified wollastonite at all ratios. There is only a peak at 1700 cm^−1^ for the sample with the highest acid-to-wollastonite ratio. The existence of this peak in the modified wollastonite samples shows that there is acid on the surface. However, there is no sign of significant interaction.

#### 3.3.5. Pimelic Acid

**Figure 9:**
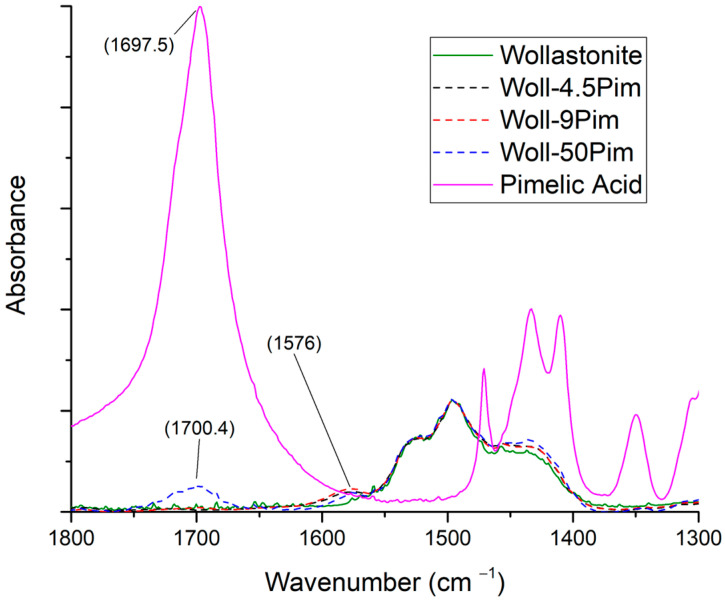
FTIR spectra of pimelic acid, wollastonite, and modified wollastonite with pimelic acid in three ratios.

In Figure 9, the spectra of pimelic acid, wollastonite, and wollastonite modified with pimelic acid at three ratios are shown in the 1800–1300 cm^−1^ range. The full spectra, at the 400–4000 cm^−1^ range, are shown in Figure S12 of the Supplementary Information file. In the case of pimelic acid, the new peak at 1580 cm^−1^ appears for all modification ratios. Nevertheless, the increase in pimelic acid did not result in a higher peak. This might be caused by the existence of a plateau that is reached at low ratios. This can be further supported since for the Woll-50 Pim sample (the sample with the highest acid to wollastonite ratio), there is a peak around 1700 cm^−1^, indicating that there is an excess of pimelic acid that did not interact with the surface of wollastonite, while the peak at 1580 cm^−1^ is similar to that of the Woll-9 Pim sample.

#### 3.3.6. Suberic Acid

**Figure 10:**
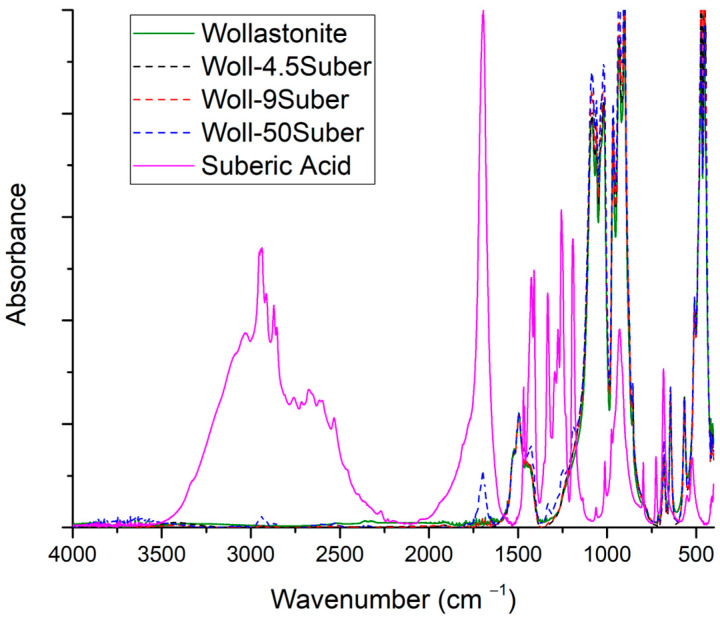
FTIR spectra of suberic acid, wollastonite, and modified wollastonite with suberic acid in three ratios.

In Figure 10, the spectra of suberic acid, wollastonite, and wollastonite modified with suberic acid at three ratios are shown. Results are similar to the glutaric acid, i.e., no significant interaction between the acid and the mineral is concluded via the comparison of the spectra.

### 3.4. Characterization Results of PP–Modified Wollastonite Drawn Fibers

Based on the FTIR results of Section 3.3, the wollastonite samples which were used for further analysis were only those presenting indications of a rather significant acid–wollastonite interaction, and from those samples, only those with the highest acid-to-wollastonite ratio. More specifically, indications of significant interactions were observed for the modification of wollastonite with myristic, maleic, malonic, and pimelic acid. Such four modified wollastonite samples were used to produce composite PP–wollastonite drawn fibers (the filler and the compatibilizer content was 2 and 4 wt%, respectively, while the drawing ratio was equal to 7, and the drawing temperature equal to 140 °C), which were characterized with tensile tests, DSC, and TGA.

**Figure 11:**
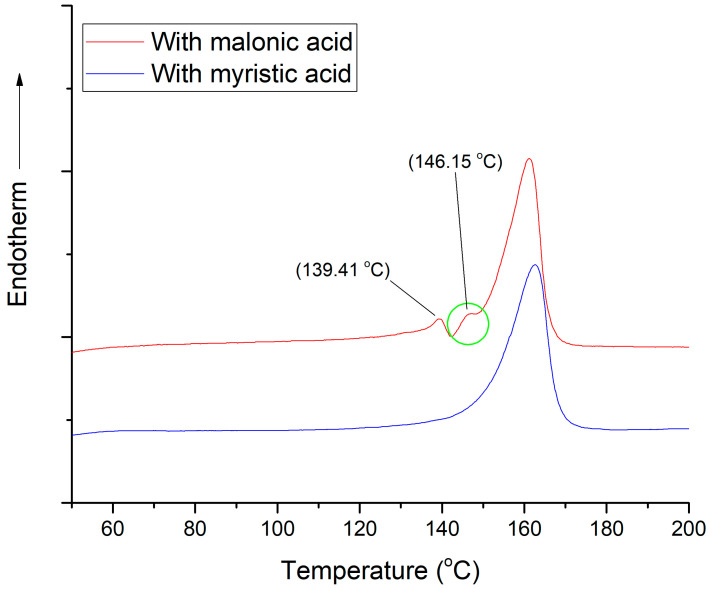
DSC curves for PP–wollastonite composites, after the second extrusion (before drawing), containing wollastonite modified with malonic acid (red, upper curve) and myristic acid (blue, lower curve).

In Figure 11, two DSC curves for composite PP–modified wollastonite samples, namely, wollastonite modified with malonic and myristic acid, after the second extrusion are presented. The curve for PP–wollastonite that is modified with myristic acid is typical for all samples, except for the samples prepared with malonic acid. In more detail, the PP composite with wollastonite that is modified with malonic acid after the second extrusion (before drawing) presents one endothermic peak around 140 °C, which is related to the melting of *β*-crystals, and one endothermic peak around 160–165 °C, attributed to the melting of *α*-crystals. At the 140–146 °C temperature range, there is also a contribution of *βα*-recrystallization. All other PP–modified wollastonite composites present only one endothermic peak around 160–165 °C, related to the melting of *α*-crystals.

Crystallinity results for the four PP–modified wollastonite composite samples are presented in Table 8. As can be seen, *β*-crystals are only present in the sample with wollastonite modified with malonic acid after the second extrusion. Based on the literature, *β*-crystals are formed if crystallization occurs in the temperature range of 115–135 °C (see introduction) and/or with the use of specific nucleating agents [7]. The results of Table 8 further support that the drawing process increases the crystallinity of the samples. Moreover, *β*-crystals are not present in all samples after drawing. In the literature, it is reported that *β*-crystals under tensile stress are converted into *α*-crystals (*βα*-recrystallization) [36]. In addition, there is no apparent crystallinity alteration between the first and the second extrusion. Typical stress–strain, DSC before and after drawing, and TGA curves are shown in Figures S13–S17, respectively, of the Supplementary Information file.

In Table 9, the characterization results for the prepared drawn PP fibers are presented. All fibers containing wollastonite, either modified or not, exhibit higher onset decomposition temperatures than the neat PP fibers. The highest onset decomposition temperature is observed for the composite fibers prepared with wollastonite that was modified with myristic acid (287 °C) and malonic acid (290 °C). However, the difference with the fibers containing unmodified wollastonite is not high (onset decomposition temperature at 280 °C).

**Figure 12:**
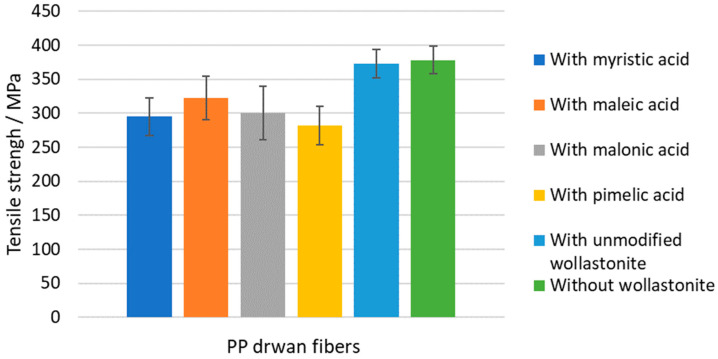
Tensile strength of PP–wollastonite drawn fibers.

**Figure 13:**
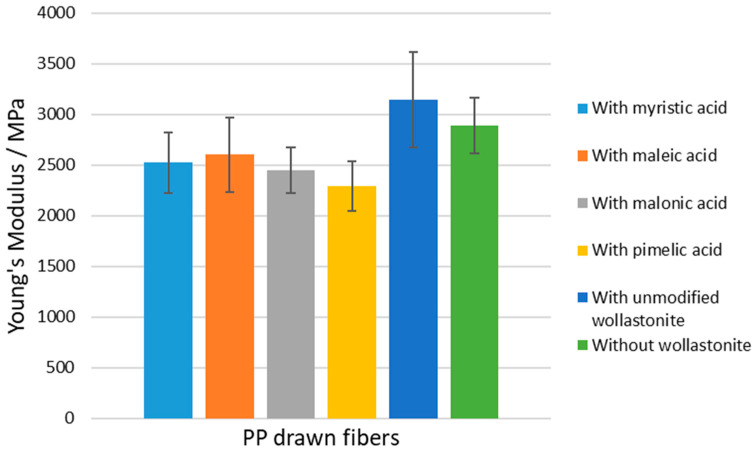
Young’s Modulus of PP–wollastonite drawn fibers.

**Figure 14:**
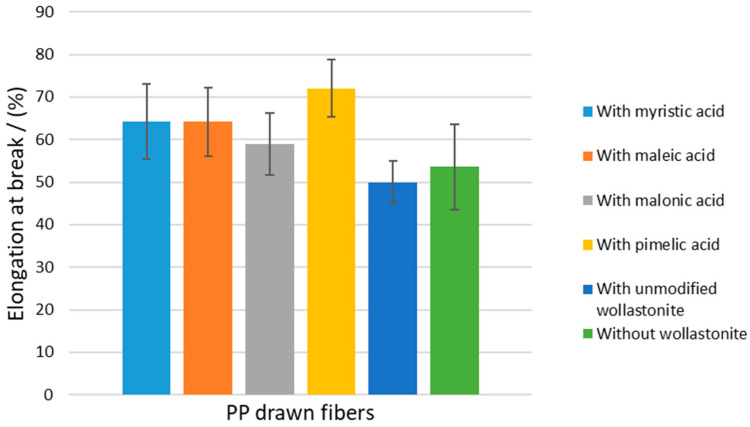
Elongation at break of PP–wollastonite drawn fibers.

Results on the mechanical properties of the prepared composite PP drawn fibers are presented in Table 9. In Figure 12, Figure 13 and Figure 14, the tensile strength, Young’s modulus, and elongation at the break of all prepared drawn fibers are shown, respectively. All samples containing modified wollastonite performed poorly, which is mainly attributed to the inadequate dispersion of the filler inside the hydrophobic polymer matrix and, to some extent, to the high crystallinity of the fibers before drawing. It is known that the high PP crystallinity (existence of *α*-crystals) before drawing results in non-homogenous drawn fibers and, consequently, leads to the deterioration of mechanical performance [36].

## 4. Conclusions

In this study, the thermal and mechanical properties of PP-modified wollastonite composite drawn fibers were investigated via DSC, TGA, and tensile tests. Modification of wollastonite was performed via organic acid treatment. Various organic acids were studied. Also, the effect of the type of solvent used to modify wollastonite with organic acids was studied. It was shown that the modification of wollastonite in a non-polar solvent, such as carbon tetrachloride, slightly enhances the adsorbed acid on the surface of the mineral, compared to ethanol. Among the six different acids that were used for the modification of wollastonite, only myristic, maleic, malonic, and pimelic acids showed that they can interact with wollastonite’s surface. In samples containing wollastonite modified with malonic acid, *β*-crystals were formed after the second extrusion. Those crystals were not present after drawing as tensile stress induces *βα*-recrystallization. In addition, the onset decomposition temperature increases by about 5–10 °C with the addition of 2% wollastonite, modified or not. Regarding mechanical properties, the composite samples exhibited poorer mechanical properties compared to the neat PP, possibly due to inadequate dispersion of the filler in the polymer matrix and, to some extent, due to the high crystallinity of the fibers before drawing. However, the crystallinity of PP–wollastonite composite fibers increased by around 10% after drawing. The use of the compatibilizer did not show any significant effect on the mechanical and thermal properties of composite drawn fibers.

Future research on this topic includes the use of less hydrophobic polymers for testing the dispersion of the investigated modified wollastonite particles and the use of particles with a less diameter-to-length ratio.

## Figures and Tables

**Table 1:** Composition of eight modified wollastonite samples (modified with myristic acid in ethanol) used for the study of the compatibilizer’s effect.

Sample Name	Myristic Acid/Wollastonite (g/g)	Wollastonite Content (%)	Compatibilizer Content (%)
0_0	0	0 ± 0	4.00 ± 0.07
0_2	0	2.00 ± 0.07	4.00 ± 0.08
1_2	0.998	2.00 ± 0.07	4.00 ± 0.08
2_2	1.974	2.00 ± 0.07	4.00 ± 0.08
0_0_NoBR (pure PP)	0	0	0
0_2_NoBR	0	2.00 ± 0.07	0
1_2_NoBR	0.998	2.00 ± 0.07	0
2_2_NoBR	1.974	2.00 ± 0.07	0

**Table 2:** Characterization results for composite drawn fibers either containing compatibilizer or not.

Sample Name	Young’s Modulus (MPa)	Tensile Strength (MPa)	Elongation at Break (%)	% Crystallinity		Onset Decomposition Temperature (°C)
				Before Drawing	After Drawing	
0_0	2888 ± 278	378 ± 20	53 ± 10	38	50	275
0_2	3140 ± 470	373 ± 20	50 ± 5	42	52	280
1_2	2584 ± 277	339 ± 23	74 ± 5	40	49	288
2_2	2646 ± 266	333 ± 9	61 ± 7	42	48	294
0_0_NoBR	2606 ± 271	353 ± 19	73 ± 15	38	47	277
0_2_NoBR	2481 ± 166	368 ± 19	60 ± 6	41	47	281
1_2_NoBR	2304 ± 153	343 ± 18	68 ± 4	40	49	287
2_2_NoBR	2459 ± 169	317 ± 15	76 ± 8	40	52	280

**Table 3:** Myristic acid to wollastonite ratio for samples modified with ethanol and carbon tetrachloride.

Sample	Solvent	Myristic Acid/Wollastonite (g/g)	Myristic Acid/Wollastonite (×10^−5^ mol/g)
1	Ethanol	0	0
2	Ethanol	0.998	4.370
3	Ethanol	1.974	8.643
4	Carbon Tetrachloride	0	0
5	Carbon Tetrachloride	1.015	4.445
6	Carbon Tetrachloride	2.028	8.882

**Table 4:** Peak ratios comparison for samples 3 and 6, which were prepared using ethanol and carbon tetrachloride as solvents, respectively.

Peak Ratios	Sample 3 (Ethanol)/ g_wol_/g_acid_	Sample 6 (Carbon Tetrachloride)/ g_wol_/g_acid_
(COO^−^, 1580 cm^−1^)/(Si–O–Si bending, 560 cm^−1^)	0.095	0.112
(COO^−^, 1580 cm^−1^)/(Si–O–Si bending, 970 cm^−1^)	0.027	0.030
(COO^−^, 1580 cm^−1^)/(Si–O vibration, 1010 cm^−1^)	0.013	0.015

**Table 5:** Composition of polypropylene–wollastonite composite drawn fibers. Wollastonite was modified with myristic acid using ethanol or carbon tetrachloride as a solvent.

Sample Name	Wollastonite Sample (See Table 3)	Wollastonite Content (%)	Compatibilizer Content (%)
0_Eth	1	2.00 ± 0.07	4.00 ± 0.08
1_Eth	2	2.00 ± 0.07	4.00 ± 0.08
2_Eth	3	2.00 ± 0.07	4.00 ± 0.08
0_CCl4	4	2.01 ± 0.07	4.00 ± 0.08
1_CCl4	5	2.00 ± 0.07	4.00 ± 0.08
2_CCl4	6	2.00 ± 0.07	4.01 ± 0.08

**Table 6:** Characterization results for polypropylene–modified wollastonite composite drawn fibers. Wollastonite was modified with myristic acid using ethanol or carbon tetrachloride as a solvent.

Sample Name	Young’s Modulus (MPa)	Tensile Strength (MPa)	Elongation at Break (%)	% Crystallinity		Onset Decomposition Temperature (°C)
				Before Drawing	After Drawing	
0_Eth	3140 ± 470	373 ± 20	50 ± 5	42	52	280
1_Eth	2584 ± 277	339 ± 23	74 ± 5	40	49	288
2_Eth	2646 ± 266	333 ± 9	61 ± 7	42	48	294
0_CCl4	3415 ± 349	342 ± 19	56 ± 6	39	50	282
1_CCl4	3576 ± 396	370 ± 19	57 ± 7	38	45	280
2_CCl4	3038 ± 391	330 ± 13	56 ± 3	39	48	285

**Table 7:** Organic acid-to-wollastonite ratios used for studying the effect of various organic acids.

Organic Acid	Organic Acid/Wollastonite (×10^−5^ mol/g)	Sample Name
Myristic acid	4.445 ± 0.005	Woll-4.5 Myr
	8.882 ± 0.005	Woll-9 Myr
	43.838 ± 0.007	Woll-50 Myr
Maleic acid	4.408 ± 0.009	Woll-4.5 Maleic
	8.858 ± 0.009	Woll-9 Maleic
	42.889 ± 0.007	Woll-50 Maleic
Malonic acid	4.507 ± 0.009	Woll-4.5 Malon
	8.977 ± 0.010	Woll-9 Malon
	50.003 ± 0.015	Woll-50 Malon
Glutaric acid	4.494 ± 0.008	Woll-4.5 Glut
	9.015 ± 0.008	Woll-9 Glut
	50.145 ± 0.012	Woll-50 Glut
Pimelic acid	4.501 ± 0.007	Woll-4.5 Pim
	8.984 ± 0.007	Woll-9 Pim
	50.004 ± 0.010	Woll-50 Pim
Suberic acid	4.488 ± 0.006	Woll-4.5 Suber
	8.974 ± 0.006	Woll-9 Suber
	50.058 ± 0.007	Woll-50 Suber

**Table 8:** Crystallinity of composite PP–modified wollastonite fibers modified with different acids.

PP Sample	Crystallinity (%)		
	After First Extrusion	After Second Extrusion	After Drawing
without wollastonite	Not measured	38	50
with unmodified wollastonite	Not measured	42	52
with wollastonite modified with myristic acid (Woll-50 Myr)	42	40	48
with wollastonite modified with maleic acid (Woll-50 Maleic)	40	38	47
with wollastonite modified with malonic acid (Woll-50 Malon)	40	37 (+2.7% *β*-crystals)	44
with wollastonite modified with pimelic acid (Woll-50 Pim)	38	38	47

**Table 9:** Decomposition temperature for samples containing unmodified and modified with different organic acids wollastonite.

PP Sample	Onset Decomposition Temperature (°C)	Young’s Modulus (MPa)	Tensile Strength (MPa)	Elongation at Break (%)
without wollastonite	275	2888 ± 278	378 ± 20	53 ± 10
with unmodified wollastonite	280	3140 ± 470	373 ± 20	50 ± 5
with Woll-50 Myr	287	2522 ± 298	295 ± 27	64 ± 9
with Woll-50 Maleic	280	2600 ± 367	322 ± 32	64 ± 8
with Woll-50 Malon	290	2447 ± 226	300 ± 39	59 ± 7
with Woll-50 Pim	283	2291 ± 247	282 ± 28	72 ± 7

## Data Availability

Not applicable.

## Supplementary Materials

The following supporting information can be downloaded at: https://www.mdpi.com/article/10.3390/polym15142986/s1, Figure S1: Typical stress–strain curves for composite drawn fibers (samples as presented in Table 1 of the main article); Figure S2: DSC curves for composite fibers before drawing (samples as presented in Table 1 of the main article); Figure S3: DSC curves for composite fibers after drawing (samples as presented in Table 1 of the main article); Figure S4: TGA curves for composite drawn fibers (samples as presented in Table 1 of the main article); Figure S5: Typical stress–strain curves for polypropylene–modified wollastonite composite drawn fibers. Wollastonite was modified with myristic acid using ethanol or carbon tetrachloride as a solvent (samples as presented in Table 5 of the main article); Figure S6: DSC curves for polypropylene–modified wollastonite composite fibers before drawing. Wollastonite was modified with myristic acid using ethanol or carbon tetrachloride as a solvent (samples as presented in Table 5 of the main article); Figure S7: DSC curves for polypropylene–modified wollastonite composite fibers after drawing. Wollastonite was modified with myristic acid using ethanol or carbon tetrachloride as a solvent (samples as presented in Table 5 of the main article); Figure S8: TGA curves for polypropylene–modified wollastonite composite drawn fibers. Wollastonite was modified with myristic acid using ethanol or carbon tetrachloride as a solvent (samples as presented in Table 5 of the main article); Figure S9: FTIR spectra of myristic acid, wollastonite, and modified wollastonite with myristic acid in three ratios (samples as presented in Table 7 of the main article); Figure S10: FTIR spectra of maleic acid, wollastonite, and modified wollastonite with maleic acid in three ratios (samples as presented in Table 7 of the main article); Figure S11: FTIR spectra of malonic acid, wollastonite, and modified wollastonite with malonic acid in three ratios (samples as presented in Table 7 of the main article); Figure S12: FTIR spectra of pimelic acid, wollastonite, and modified wollastonite with pimelic acid in three ratios (samples as presented in Table 7 of the main article); Figure S13: Typical stress–strain curves for composite drawn PP–modified wollastonite fibers modified with different acids (samples as presented in Table 7 of the main article); Figure S14: DSC curves for composite PP–modified wollastonite fibers modified with different acids after 1st extrusion; Figure S15: DSC curves for composite PP–modified wollastonite fibers modified with different acids after 2nd extrusion; Figure S16: DSC curves for composite PP–modified wollastonite fibers modified with different acids after drawing; Figure S17: TGA curves for composite drawn PP–modified wollastonite fibers modified with different acids (samples as presented in Table 7 of the main article).
