# Supplementary material for: Effect of the Filler Modification on the Thermal and Mechanical Properties of Composite Polypropylene/Wollastonite Drawn Fibers
Source: Polymers (Basel). 2023 Jul 8;15(14):2986. doi: 10.3390/polym15142986 (PMC10383438; doi:10.3390/polym15142986)
Supplement: Supplementary file 1 [file polymers-15-02986-s001.zip › polymers-2439550-supplementary.pdf]

polymers

Supplementary Information for

Effect of the Filler Modification on the Thermal and Mechanical Properties of Composite Polypropylene/Wollastonite Drawn Fibers

Konstantinos Leontiadis [1], Dimitris S. Achilias [2] and Ioannis Tsivintzelis [1,*]

[1] Department of Chemical Engineering, Aristotle University of Thessaloniki, 54124 Thessaloniki, Greece; leontiad@cheng.auth.gr
[2] Department of Chemistry, Aristotle University of Thessaloniki, 54124 Thessaloniki, Greece; axilias@chem.auth.gr
* Correspondence: tioannis@cheng.auth.gr

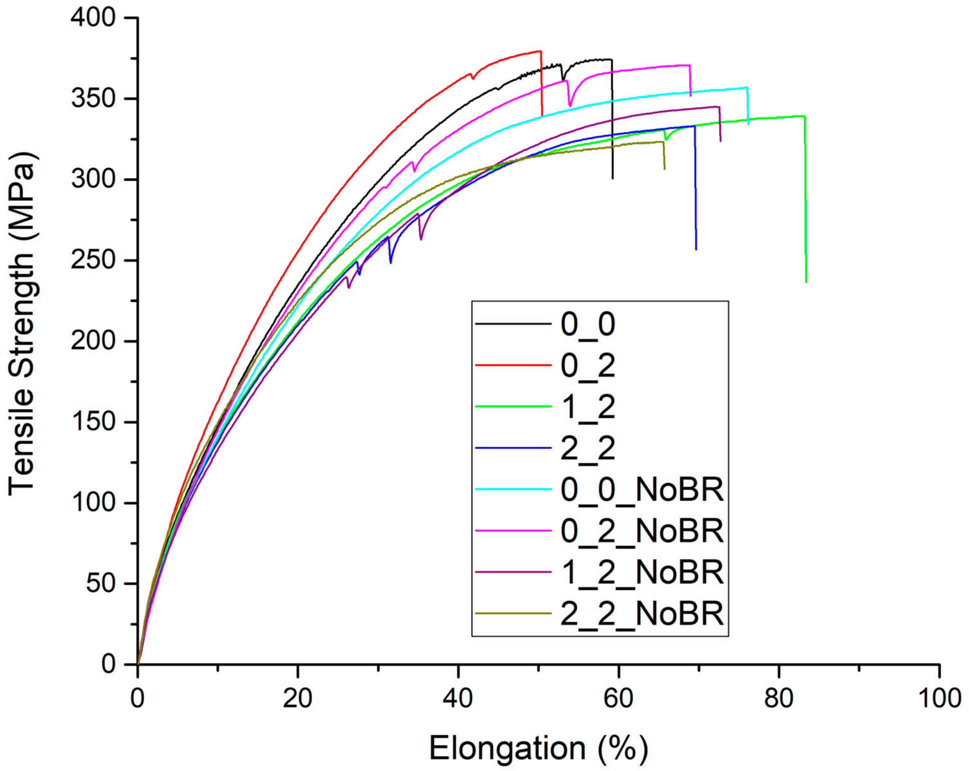

Figure S1. Typical stress – strain curves for composite drawn fibers (samples as presented in Table 1 of the main article).

Citation: Leontiadis, K.; Achilias, D.S.; Tsivintzelis, I. Effect of the Filler Modification on the Thermal and Mechanical Properties of Composite Polypropylene/ Wollastonite Drawn Fibers. *Polymers* **2023**, *15*, 2986. https://doi.org/ 10.3390/polym15142986

Academic Editors: Vincenzo Fiore and Łukasz Klapiszewski

Received: 23 May 2023
Revised: 21 June 2023
Accepted: 5 July 2023
Published: 8 July 2023

Copyright: © 2023 by the authors. Licensee MDPI, Basel, Switzerland. This article is an open access article distributed under the terms and conditions of the Creative Commons Attribution (CC BY) license (https://creativecommons.org/licenses/by/4.0/).

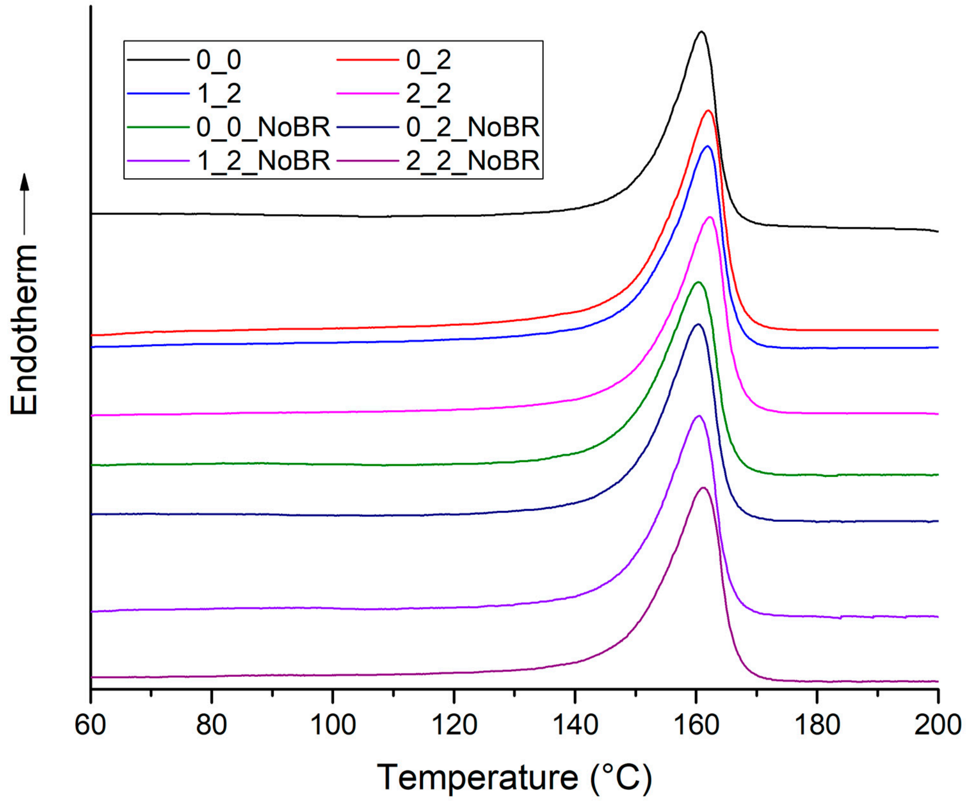

Figure S2. DSC curves for composite fibers before drawing (samples as presented in Table 1 of the main article).

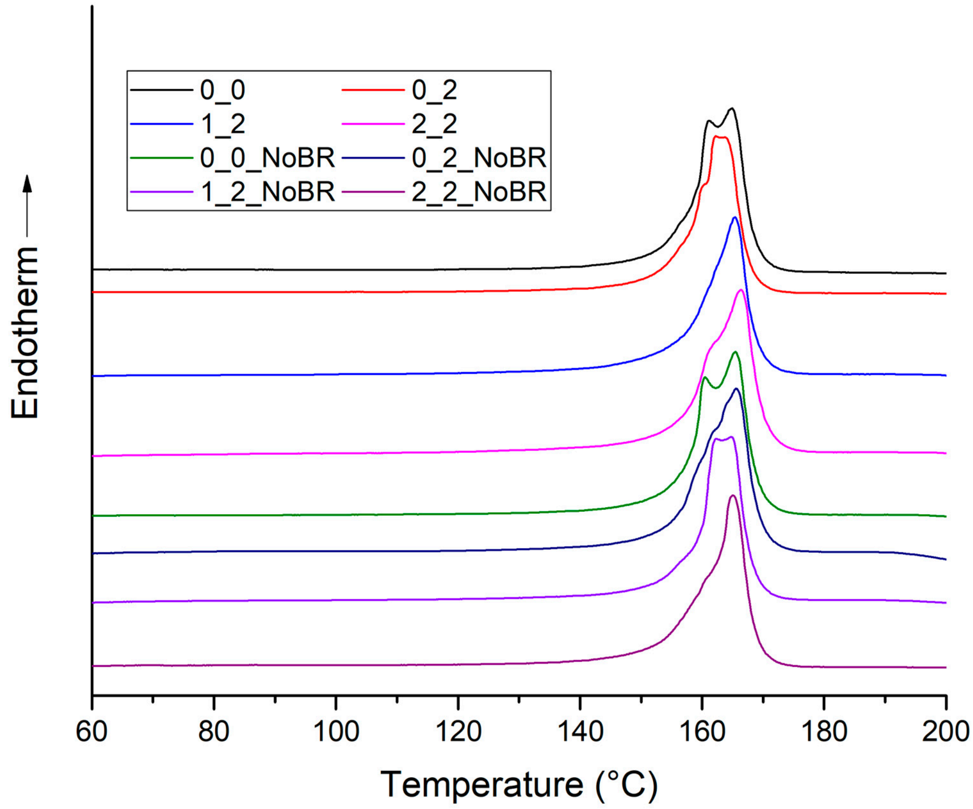

Figure S3. DSC curves for composite fibers after drawing (samples as presented in Table 1 of the main article).

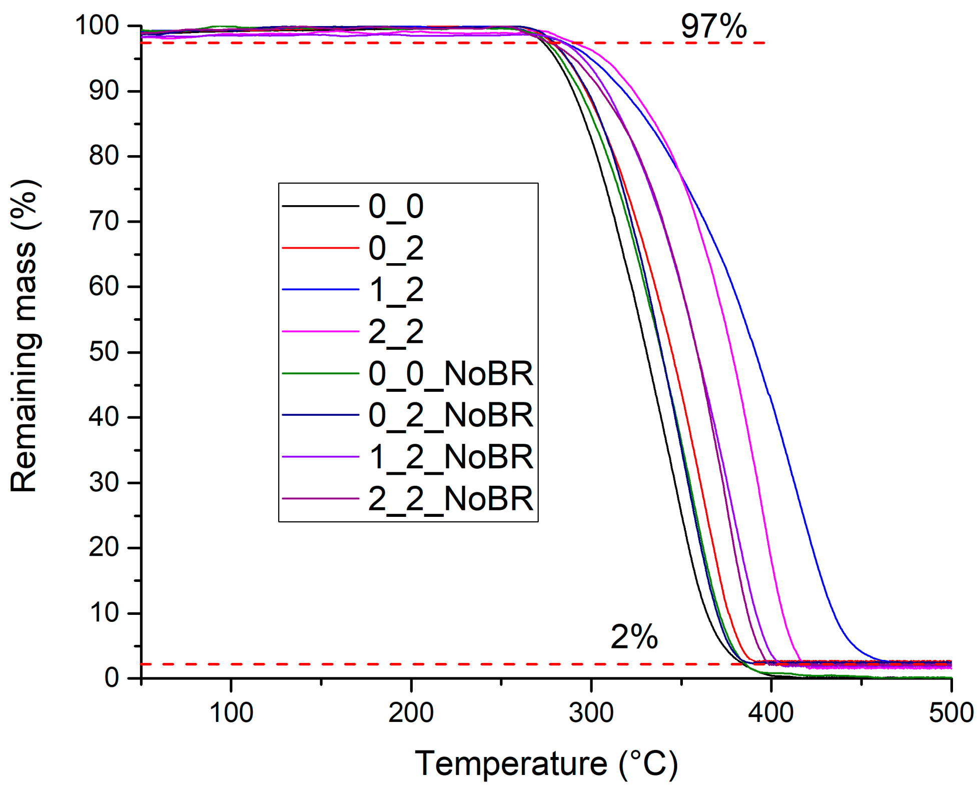

Figure S4. TGA curves for composite drawn fibers (samples as presented in Table 1 of the main article).

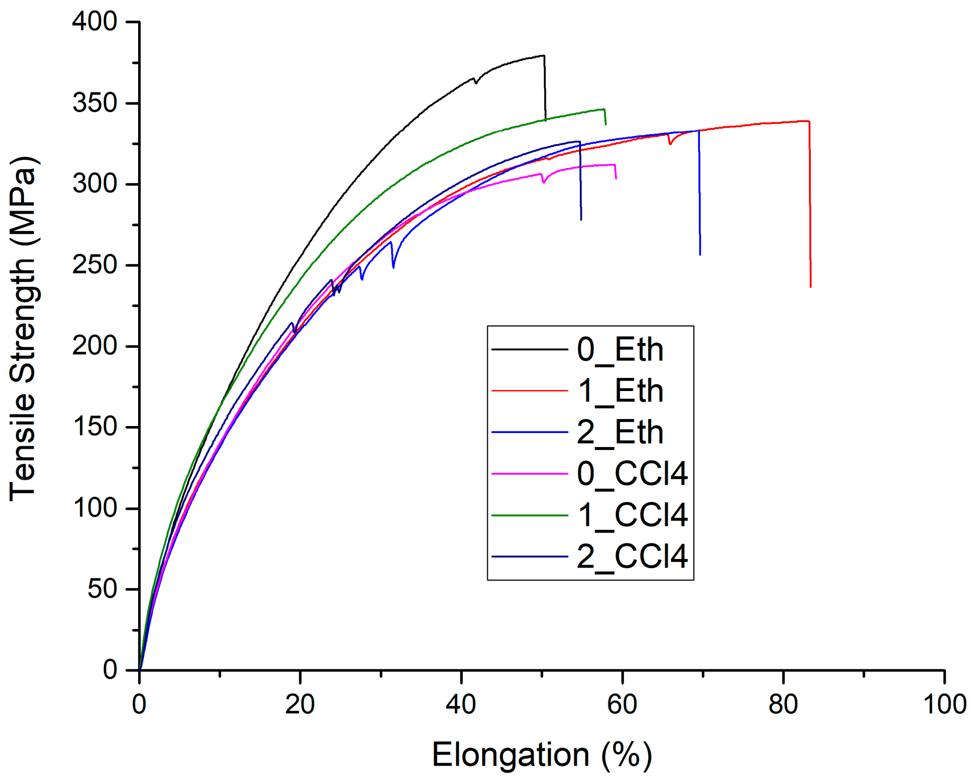

Figure S5. Typical stress – strain curves for polypropylene – modified wollastonite composite drawn fibers. Wollastonite was modified with myristic acid using ethanol or carbon tetrachloride as a solvent (samples as presented in Table 5 of the main article).

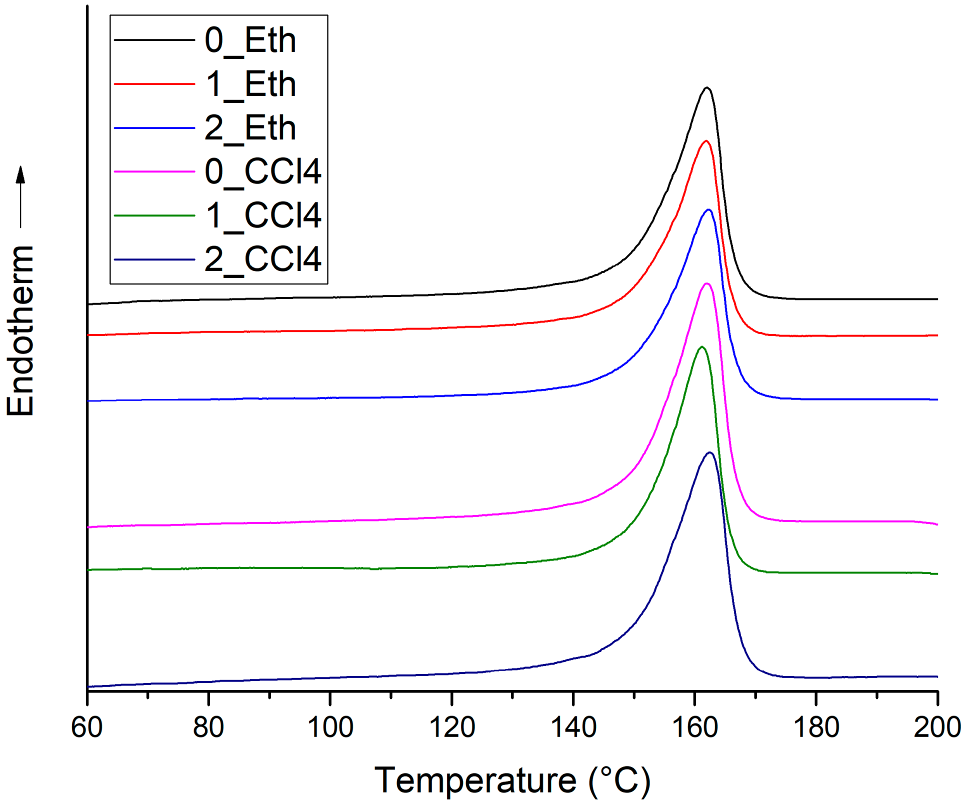

Figure S6. DSC curves for polypropylene – modified wollastonite composite fibers before drawing. Wollastonite was modified with myristic acid using ethanol or carbon tetrachloride as a solvent (samples as presented in Table 5 of the main article).

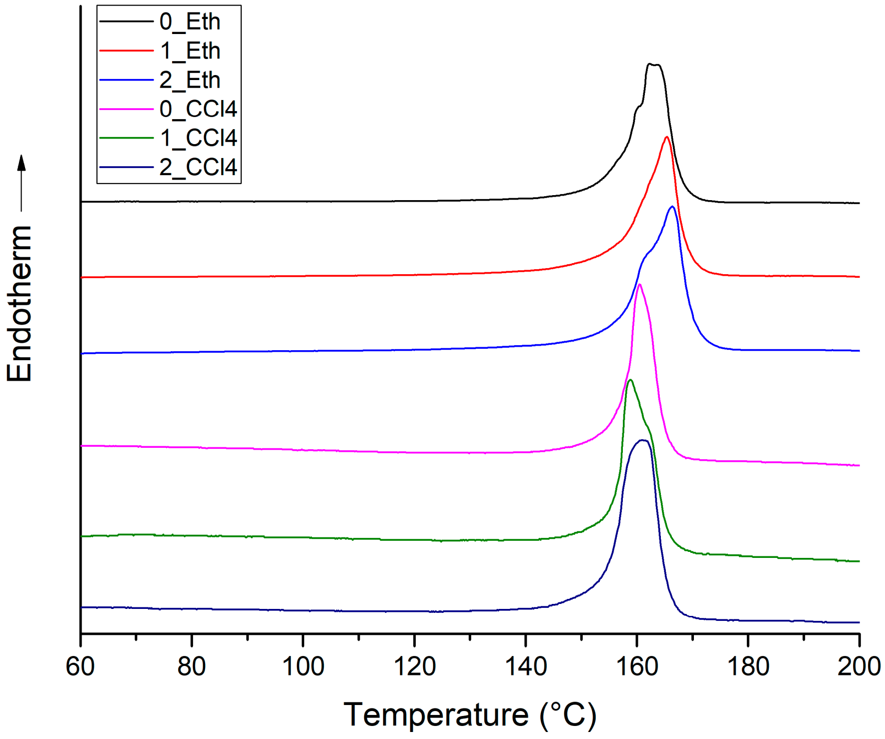

Figure S7. DSC curves for polypropylene – modified wollastonite composite fibers after drawing. Wollastonite was modified with myristic acid using ethanol or carbon tetrachloride as a solvent (samples as presented in Table 5 of the main article).

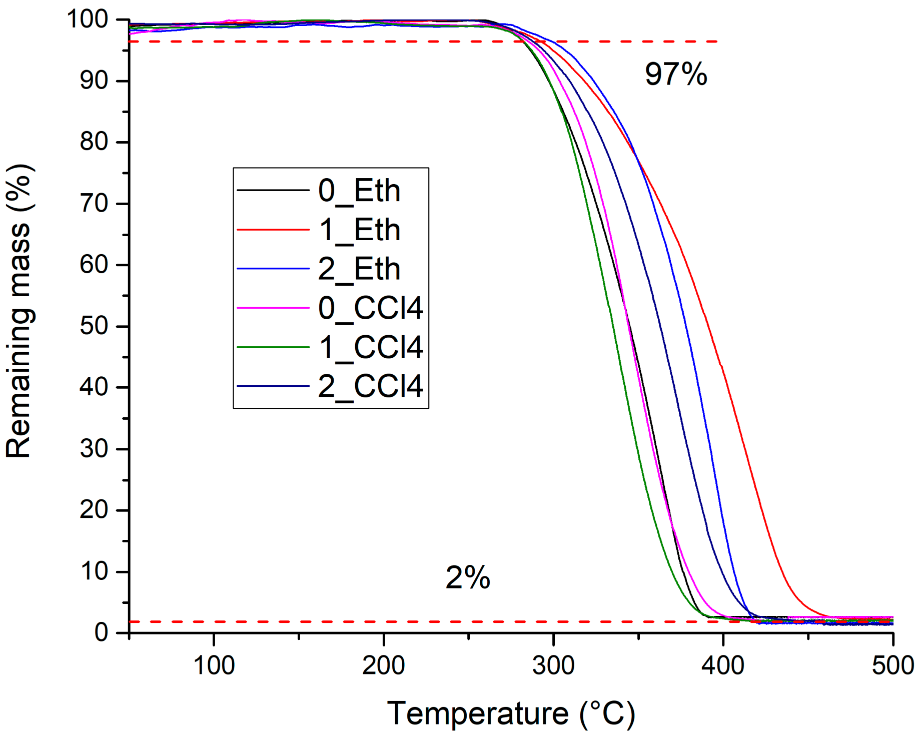

Figure S8. TGA curves for polypropylene – modified wollastonite composite drawn fibers. Wollastonite was modified with myristic acid using ethanol or carbon tetrachloride as a solvent (samples as presented in Table 5 of the main article).

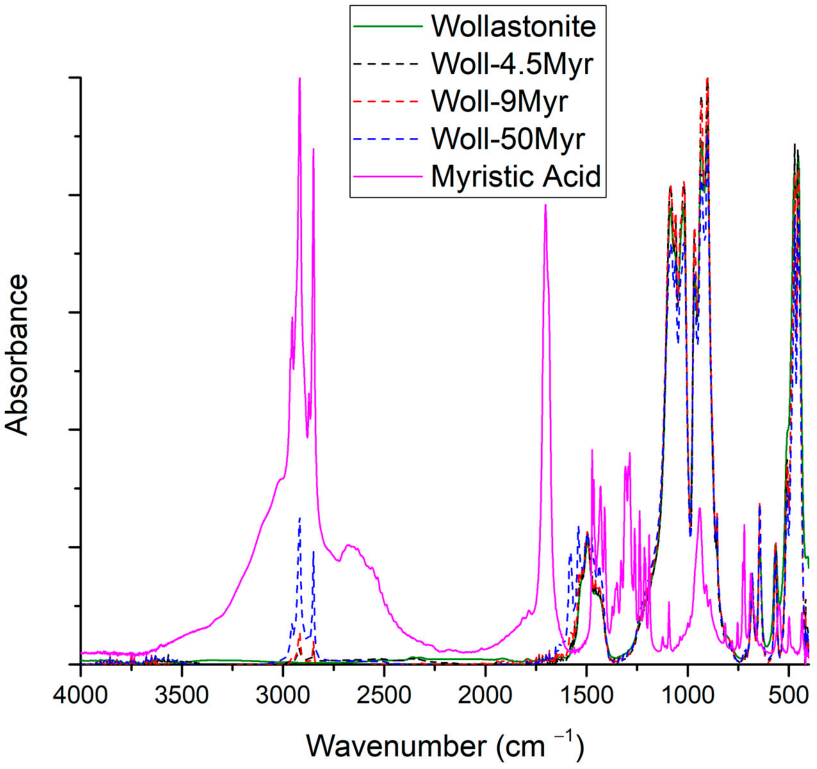

Figure S9. FTIR spectra of myristic acid, wollastonite, and modified wollastonite with myristic acid in three ratios (samples as presented in Table 7 of the main article).

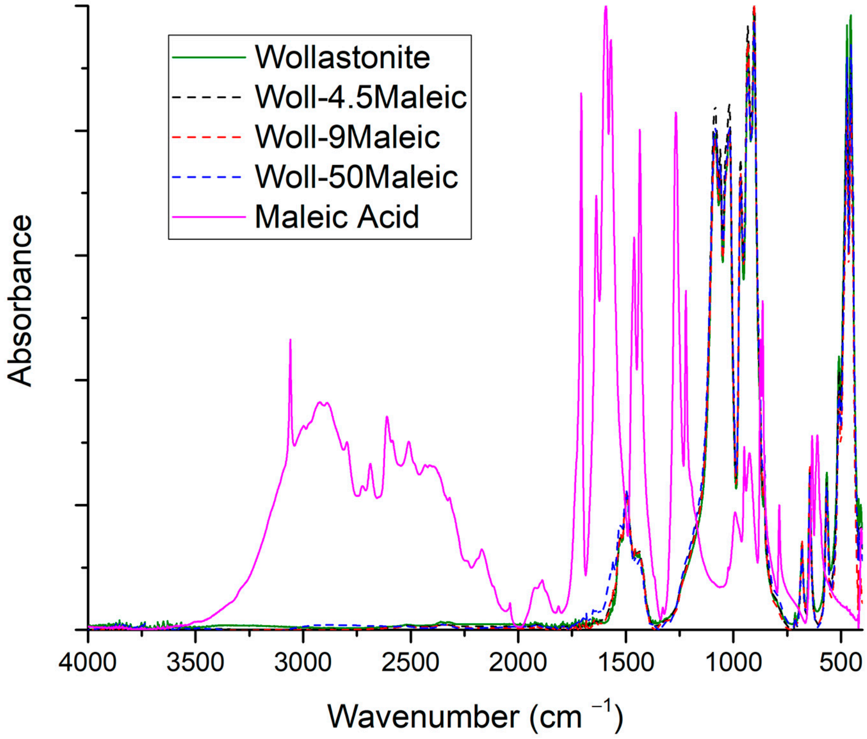

Figure S10. FTIR spectra of maleic acid, wollastonite, and modified wollastonite with maleic acid in three ratios (samples as presented in Table 7 of the main article).

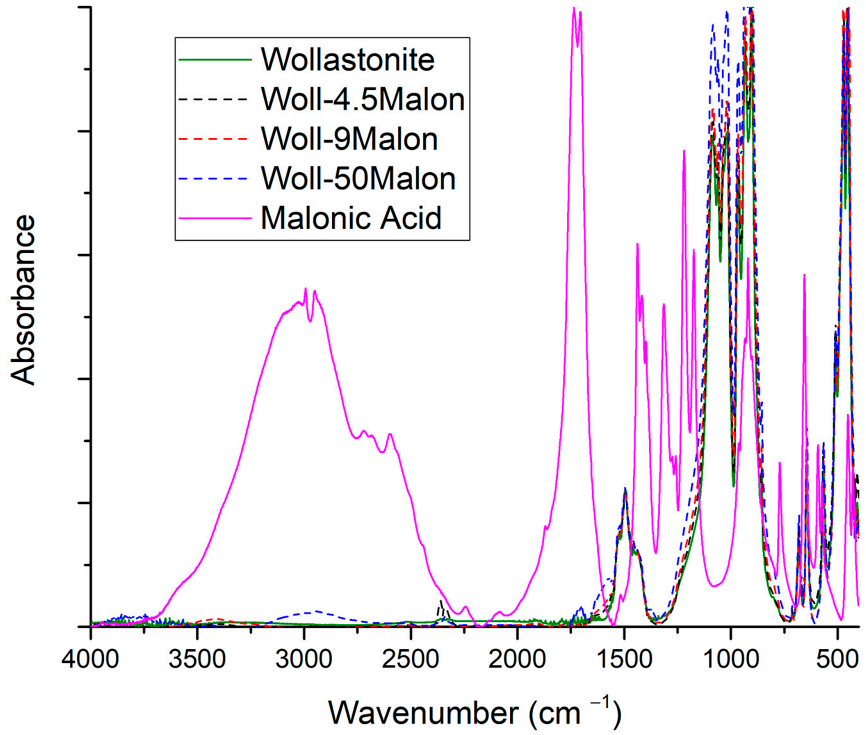

Figure S11. FTIR spectra of malonic acid, wollastonite, and modified wollastonite with malonic acid in three ratios (samples as presented in Table 7 of the main article).

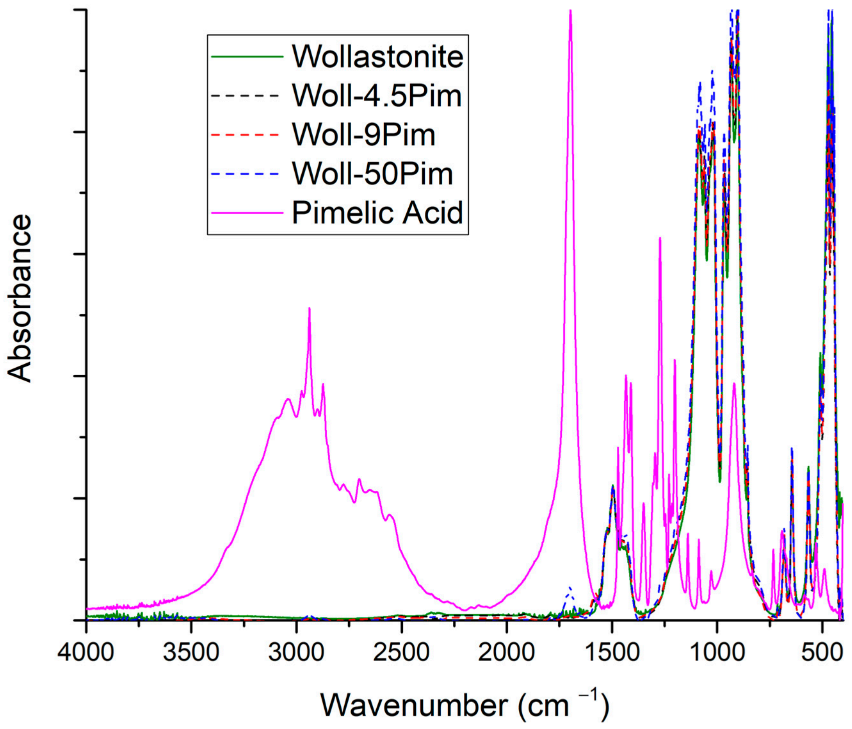

Figure S12. FTIR spectra of pimelic acid, wollastonite, and modified wollastonite with pimelic acid in three ratios (samples as presented in Table 7 of the main article).

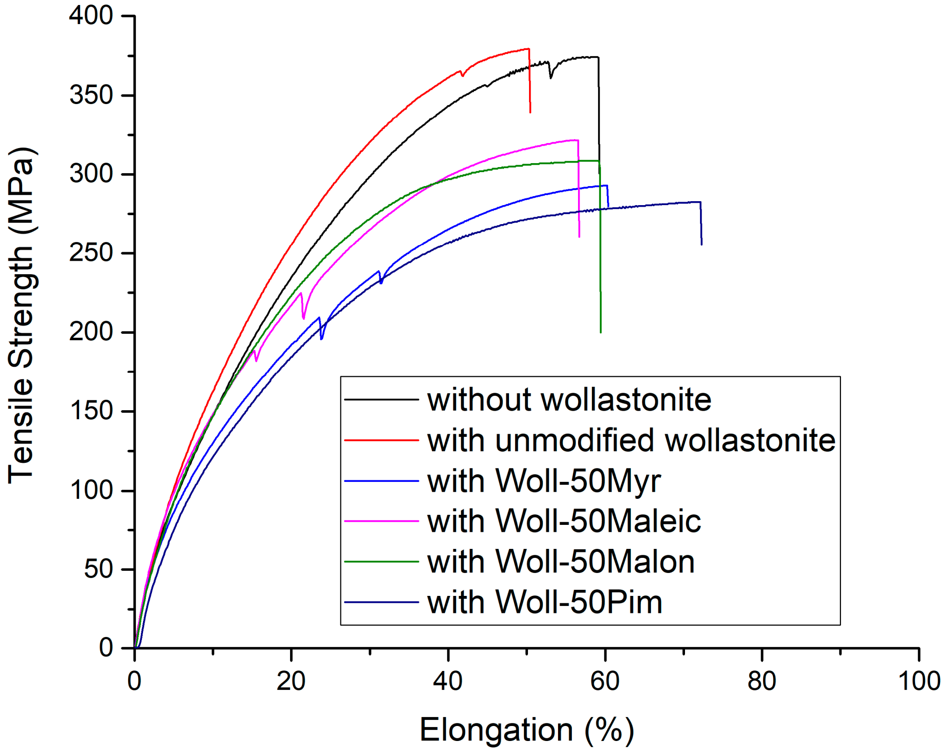

Figure S13. Typical stress – strain curves for composite drawn PP – modified wollastonite fibers modified with different acids (samples as presented in Table 7 of the main article).

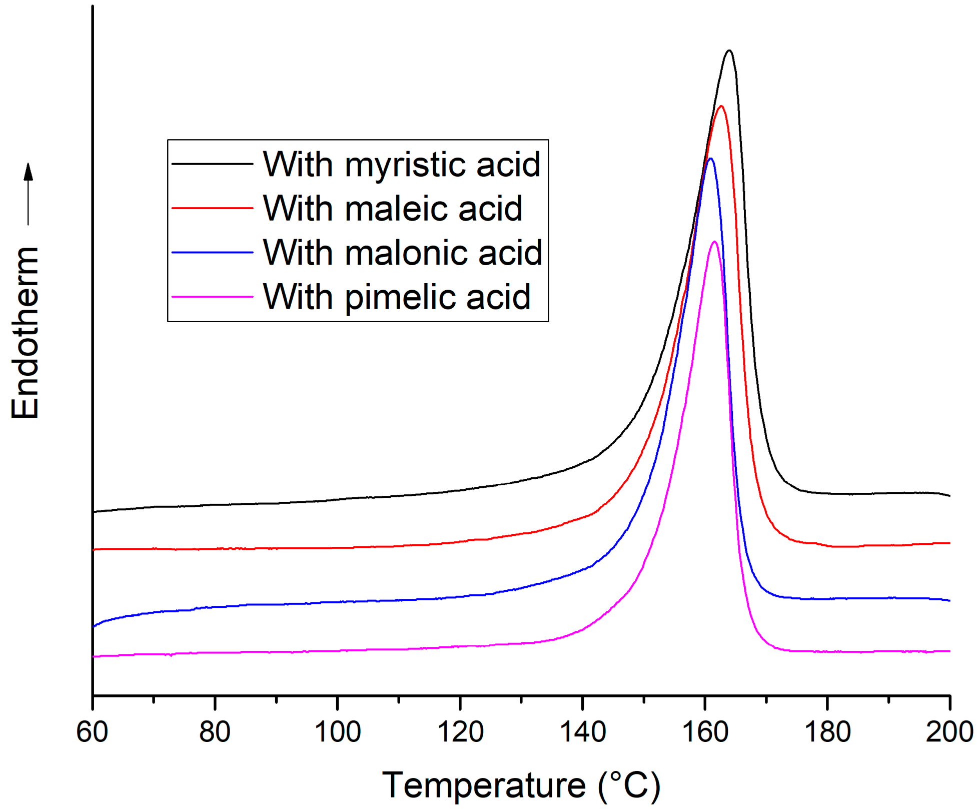

Figure S14. DSC curves for composite PP – modified wollastonite fibers modified with different acids after 1st extrusion.

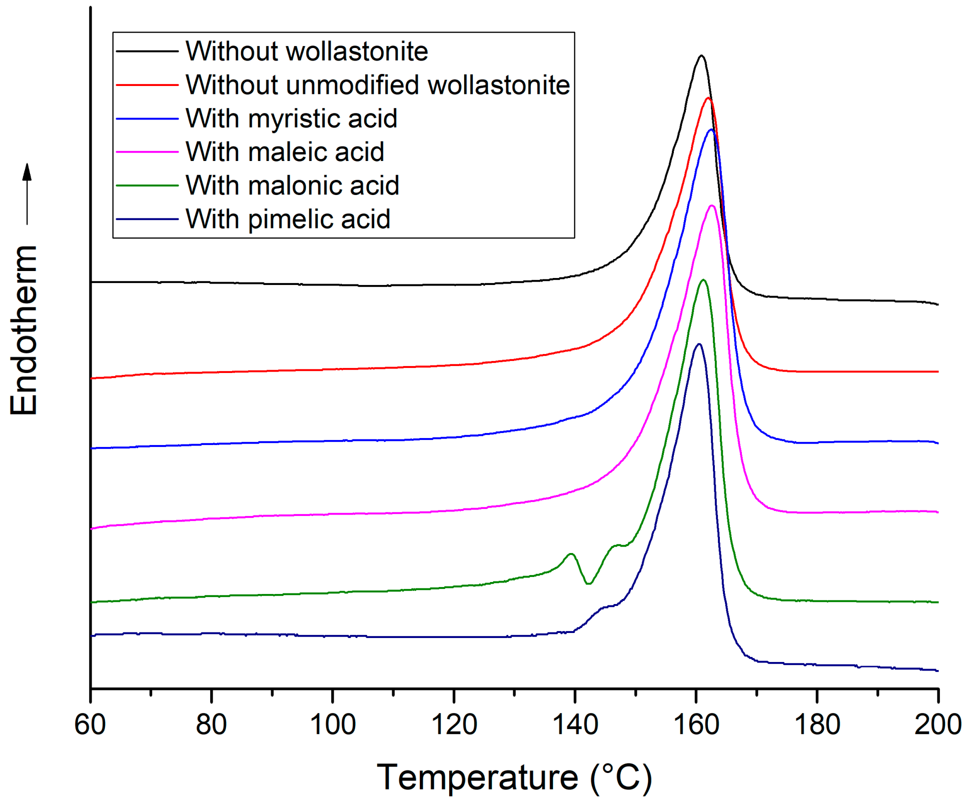

Figure S15. DSC curves for composite PP – modified wollastonite fibers modified with different acids after 2nd extrusion (before drawing).

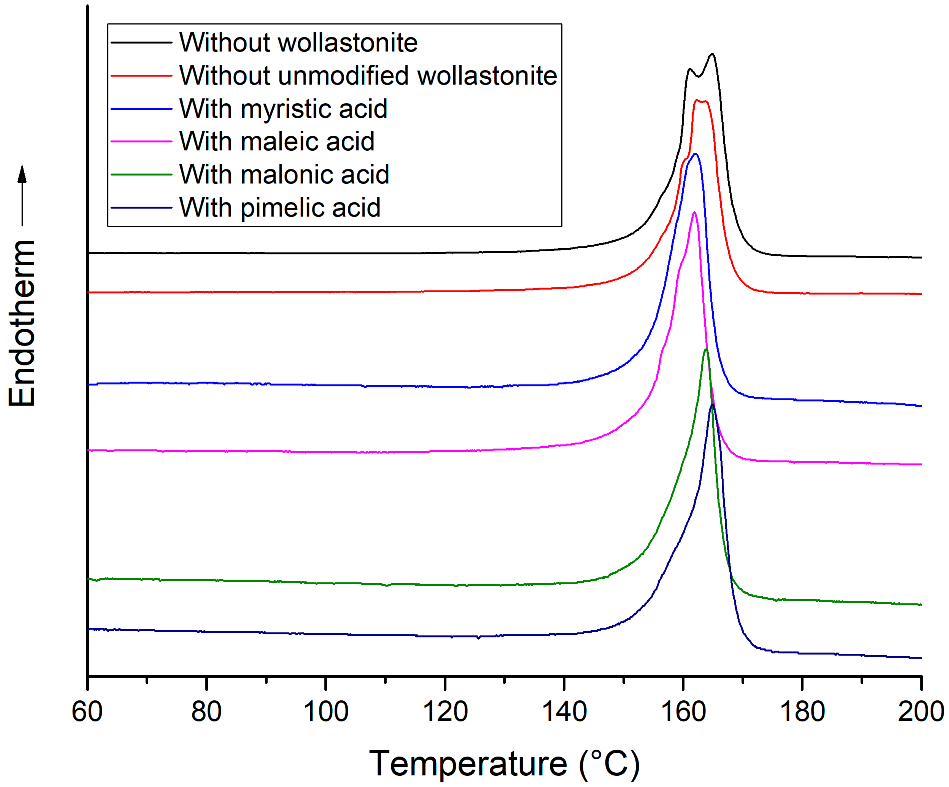

Figure S16. DSC curves for composite PP – modified wollastonite fibers modified with different acids after drawing.

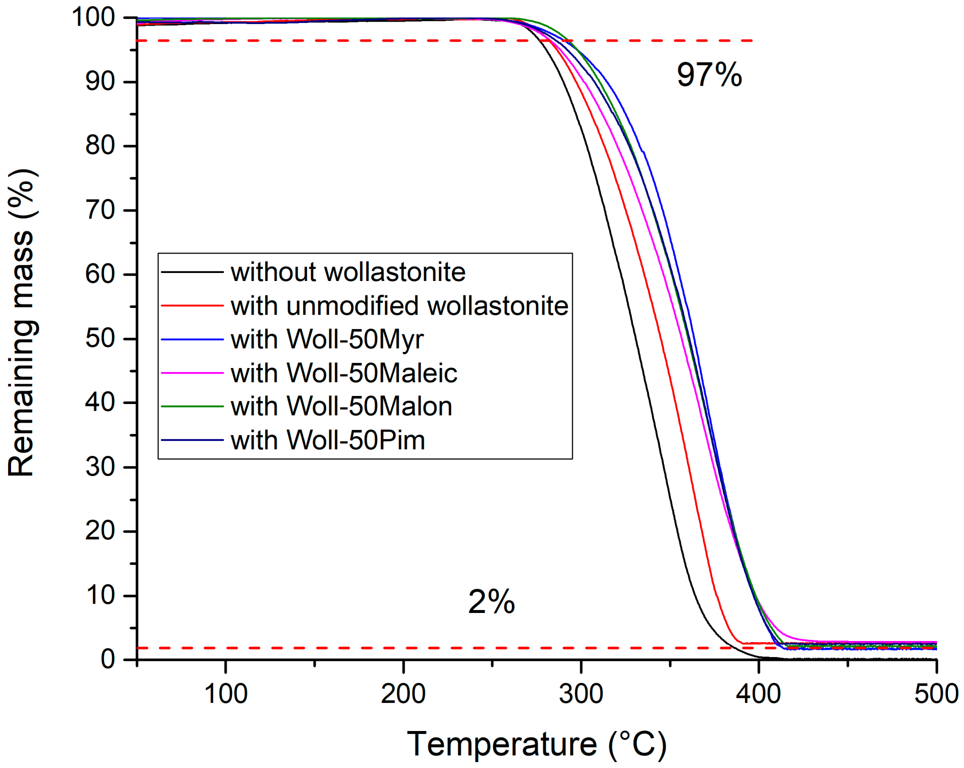

Figure S17. TGA curves for composite drawn PP – modified wollastonite fibers modified with different acids (samples as presented in Table 7 of the main article).

Disclaimer/Publisher's Note: The statements, opinions and data contained in all publications are solely those of the individual author(s) and contributor(s) and not of MDPI and/or the editor(s). MDPI and/or the editor(s) disclaim responsibility for any injury to people or property resulting from any ideas, methods, instructions or products referred to in the content.
